# Comment on “Kennedy class III and IV dental arches”

**DOI:** 10.1590/0103-644020256531

**Published:** 2025-12-08

**Authors:** Hinpetch Daungsupawong, Viroj Wiwanitkit

**Affiliations:** 1 Private Academic Consultant, Phonhong, Vientiane Lao People's Democratic Republic; 2 Department of Research Analytics, Saveetha Dental College and Hospitals, Saveetha Institute of Medical and Technical Sciences Saveetha University, Chennai India

**Figure:**
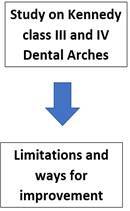

Dear Editor,

We would like to comment on “Kennedy class III and IV dental arches: Trueness analysis of digitization methods and 3D-printing step [1].” The purpose of this study was to evaluate the correctness of Kennedy class III and IV digitized teeth that were 3D printed using stereolithography technology in an in vitro setting. Reference molds of Kennedy class III and IV teeth were created and constructed using CAD/CAM, and parameters such as occlusocervical, interarch, and edentulous space were assessed using intraorbital (IOS), extraorbital (EOS), and cone-beam computed tomography (CBCT). A total of 60 experimental sets were digitized and 3D printed using the digital data. The experimental groups were separated into three groups (10 pieces each). The measurements were repeated on the experimental specimens and analyzed with a two-way ANOVA and a post-Bonferroni test. The error measurements revealed that class 4 had the most significant error compared to class 3, and this error increased after 3D printing. The most significant inaccuracy in edentulous spaces was discovered when CBCT was digitized and turned into 3D models.

Although this study was well-designed in terms of assessing the accuracy of both types of dental molds, there are some drawbacks to the technique. For example, using only 60 samples may not be enough to represent the diversity of different dental conditions, or evaluating a constrained laboratory environment (in vitro) that cannot imitate the use in real patients. Furthermore, the error measuring approach is inaccurate because external factors, such as printing or scanning equipment settings, can lead to discrepancies in results. This is something to think about in further analysis.

In this study, statistical tools such as ANOVA and the Post-Bonferroni test were used to allow for a good comparison of results between groups. However, relying solely on these two tests may not be sufficient to investigate the elements that could have a substantial impact on the study outcomes, such as issues related to 3D printing, including printer accuracy, printing settings, or factors affecting prototype development in the case of class 4.

To promote creativity and future research directions, the study's scope should be expanded to include real-world patient testing. Or in a more real-world application setting. Furthermore, comparing and analyzing the long-term results of various 3D printing technologies will be critical in determining the strengths and limitations of each approach in real-world applications.
